# Comparison of the Outcomes of Three Different Nutritional Supports in Patients with Oral and Maxillofacial Malignant Tumors following Surgery

**DOI:** 10.1155/2018/5627141

**Published:** 2018-11-01

**Authors:** Chen Zou, Xuan Zhou, Shuhan Zhuang, Guowei Huang, Hongwu Wang

**Affiliations:** ^1^Department of Nutrition, Tianjin Stomatological Hospital, No. 75 Dagu Road, Heping District, Tianjin 300041, China; ^2^Department of Nutrition and Food Science, School of Public Health, Tianjin Medical University, No. 22 Qixiangtai Road, Heping District, Tianjin 300070, China; ^3^Chinese Internal Medicine, Graduate School, Tianjin University of Traditional Chinese Medicine, No. 10 Boyanghu Road, Jinghai District, Tianjin 301617, China; ^4^Department of Public Health, Tianjin University of Traditional Chinese Medicine, No. 10 Boyanghu Road, Jinghai District, Tianjin 301617, China

## Abstract

*Objective. *This study aimed to compare the physical and mental states and the clinical effects of parenteral nutrition combined with enteral nutrition (PN+EN), total enteral nutrition (TEN), and total parenteral nutrition (TPN) after surgery in patients with maxillofacial malignant tumors.* Methods.* A total of 112 patients were divided into three groups, with 58, 33, and 21 patients in the PN+EN, TPN, and TEN groups, respectively. The psychological survey contained the Faces Pain Scale-Revised (FRS-R), visual analog scale (VAS), numerical rating scale (NRS), Hamilton anxiety rating scale (HAMA), and short-form 36 health survey questionnaire (SF-36). Spirit symptoms, length of hospital stay, nutritional assessments, and related biochemical indices were recorded and compared.* Results.* The traditional Chinese medicine (TCM) symptoms of anxiety and dysphoria were least frequently identified in the TPN group. The levels of lymphocytes, hemoglobin (HB), albumin (ALB), and prealbumin (PA) were significantly higher in the PE+EN group, whereas white blood cell count, neutrophil count, HB, PA, and ALB were significantly lower in the TPN group. Better psychological scores were observed in the TPN group. The PE+EN group had a shorter length of stay and higher SGA categories. Potassium, sodium, and chlorine levels were significantly lower in the TEN group (all* P* < 0.05).* Conclusions.* As an auxiliary method, TCM symptoms can help to identify spirit disequilibrium earlier and are associated with blood indices. Without the consideration of cost and long length of hospital stay, patients in the TPN group had the best mental status, with PN+EN therapy being an alternative.

## 1. Introduction

The application of vascular pedicled skin flap transfer in the repair of jaw defects in patients with maxillofacial malignancy can restore jaw continuity and facial shape but may also help patients to chew, swallow, and correctly pronounce words [1].

However, operation time for flap transplantation is long and vasoconstriction of the vascular smooth muscle caused by the stimulation of the epicardial membrane during the operation, postoperative pain, cold, and other factors lead to the stimulation of the sympathetic nervous system, causing cvasospasm, tissue and vascular injury, hypercoagulable blood, and keeping head motionless 1-3 days after the operation. Therefore, the quality of life of patients is reduced, especially in the first three days. Thus, the treatment of oral and maxillofacial malignant tumors includes not only cure and survival rates but also the mental state of the patients [2].

The modern medical model has been transformed to a biological-psychosocial-medical model. The resection of the oropharyngeal and laryngopharynx tumors destroys the body's original physiological structure, thus making it more difficult to place the tube. Studies have shown that patients with head and neck tumors face a significant psychological burden in the perioperative period [3, 4]

The head is the commonly exposed body part in humans and plays an important role in individual image; however, the side effects of malignant oral and maxillofacial tumor treatment may result in barriers to basic functions such as speaking and swallowing [5] and as patients in the intensive care period cannot express their wishes in a timely manner, passive acceptance of a gastric tube may occur [6, 7]. The presence of a gastric tube may have a greater negative impact on mental health status and patients may feel anxious, depressed, and even suicidal [8].

Moreover, in the intensive care period, psychological questionnaires cannot be used to assess patients because of their poor physical and mental states. However, this period is crucial for the formulation and adjustment of specific treatment plans. In traditional Chinese medicine (TCM) theory, the exterior symptoms and signs may reflect the fundamental pathology of the internal organs. In other words, the status of the internal organs can be determined by observing exterior symptoms such as the complexion or the tongue [9, 10]. The TCM theory of essence, qi, and spirit indicates that expression, mood, and speech reflect mental and physical health [11, 12]. Therefore, the present study used TCM as an auxiliary method by observing the color and luster of the complexion and the symptoms of spirit disequilibrium.

Specific psychological survey methods were used to evaluate the social support, anxiety scale, survival satisfaction, and life happiness seven days after the surgery when the physical state of patients generally improves.

Correct nutritional support can help patients improve their nutritional status, prevent complications, facilitate recovery from inflammation, increase the survival rate of the flap, and shorten the hospitalization time [13].

To identify and solve problems and to provide guidance in a timely manner, it is necessary to adjust the patient's feeding program according to patient blood indices and spirit symptoms. The present study assessed the effects of different nutritional support in patients with maxillofacial malignant tumor using both traditional measures and TCM symptoms to evaluate patient feelings and mental status.

## 2. Methods

### 2.1. Study Population

A total of 112 patients were treated by the Department of Oral and Maxillofacial Surgery at the study hospital between March 2016 and December 2017.

The inclusion criteria were ages between 40 and 70 (the age range with a high incidence of maxillofacial malignancy), an initial nutritional assessment report score < 3.0 points, and provision of informed consent. The operations often involved jaw and tumor resection, neck dissection, peroneal musculocutaneous flap preparation, and peroneal musculocutaneous flap transfer repair.

The exclusion criteria included the presence of severe endocrine and metabolic diseases or other malignancies, blood system diseases, chronic cardiopulmonary insufficiency, severe liver and kidney dysfunction, abnormal intraoperative bleeding, or the occurrence of severe postoperative complications.

This study was approved by the Independent Ethics Committee of the study hospital (no. TSHhMEC2017002). All patients provided informed consent. This study was registered in the Chinese Clinical Trial Registry (http://www.chictr.org.cn) as ChiCTR-OPC-17013869.

### 2.2. Nutritional Support

The 112 patients were divided into three groups by the nutrition support team (NST) according to the nutritional support they received:

(1) Parenteral nutrition combined with enteral nutrition (PN+EN) group: after surgery in the head, the head was immobilized for 48 to 72 hours. Between one and three days postoperatively, PN was provided as follows: 20-25 kcal/(kg·d) with a glucose-to-lipid ratio of 6:4 or 5:5; calorie-to-nitrogen ratio of 120-150:1; and vitamins, trace elements, and electrolytes as needed. We added insulin based on patients' blood glucose levels, with a glucose-to-insulin ratio of 8-10:1 g/U. If the patient had diabetes, the glucose-to-insulin ratio was 4-5:1 g/U. PN + EN was administered four days after the operation and the proportion of PN was decreased gradually. The EN dosage was the standard 20 kcal/(kg·d) at a drip rate of 50 ml/h. If no reflux, diarrhea, abdominal distension, or other gastrointestinal side effects occurred, after 2-3 days, EN was injected continuously at 25 kcal/(kg·d) at a drip rate of 100 ml/h.

(2) Total enteral nutrition (TEN) group: throughout the postsurgical period, a high-energy nutrient mixture (1 kcal/ml, Nedicia) was administered via continuous drip through a nasal feeding tube with an initial drip rate of 50 ml/h. If no reflux, diarrhea, abdominal distension, or other gastrointestinal side effects occurred, 2 to 3 days later the drip rate was increased to 100 ml/h.

(3) Total parenteral nutrition (TPN) group: several patients could not receive nasal feeding due to the location or nature of their wounds and were given the corresponding 20-25 kcal/(kg·d) and increased calories based on their condition [14].

### 2.3. Psychological Status Assessment

The psychological surveys administered after maxillofacial surgery included the Faces Pain Scale-Revised (FRS-R) [15], visual analog scale (VAS) [16], numerical rating scale (NRS) [17, 18], Hamilton anxiety rating scale (HAMA) [19], oral-facial body dysmorphic disorder self-scale (OFBDDSS) [20, 21], self-overall appearance evaluation scores [22], and short-form 36 health survey questionnaire (SF-36) [23, 24]. The surveys were conducted seven days after surgery by the NST. Higher scores indicated poorer patient mental state and feelings.

### 2.4. Biochemical Indices

Blood indices were assessed one and seven days after surgery, including measurement of the circulating levels of lymphocytes (LYM), hemoglobin (HB), albumin (ALB), and prealbumin (PA) and white blood (WBC) and neutrophil (NE) counts. Potassium (K), sodium (NA), and chlorine (CL) levels were measured and compared between groups 13 days after surgery.

### 2.5. Nutritional Status Assessment

Scored Patient-Generated Subjective Global Assessment (PG-SGA) was performed after surgery. Part A was completed by the patients, including a history questionnaire (weight assessment, dietary intake, symptoms, activities and functions, etc., in four parts); Part B included the disease and its relationship to nutritional needs (age score added); Part C included metabolic requirements; and Part D included a physical examination. The four parts are summed, with scores of 0-1 indicating no need for nutritional intervention and routine nutritional status; 2-3 points indicates the need for patient and family education guidance by dietitians, nurses or physicians, and drug intervention appropriate for symptoms and laboratory examination; 4-8 points indicate the need for nutrition intervention and treatment of symptoms; and more than 9 points indicates an urgent need to improve the symptoms of the treatment measures and to provide appropriate nutrition support. The qualitative evaluation of the rating results includes 0-1 points for good nutrition (A); 2-8 points for moderate malnutrition (B); and 9 points for severe malnutrition (C) [25, 26].

### 2.6. TCM Symptoms

The symptoms of spirit disequilibrium including observed anxiety, dysphoria, restlessness, delirious speech, and intolerance were observed. Physical condition was assessed based on pale complexion and skin with or without luster.

### 2.7. Length of Hospitalization

The length of hospitalization was defined as the total number of days from hospital admission to discharge after the wound had healed well and the sutures were removed.

### 2.8. Statistical Analyses

Shapiro-Wilk and Levene's tests were used to assess the normality and homogeneity of the data. Continuous variables were expressed as means and standard deviations. One-way analysis of variance (ANOVA) or Kruskal-Wallis tests were used to compare data between groups. Chi-squared or Fisher's exact tests were carried out to assess dichotomous variables from the three groups. The pairwise comparisons among the means were performed using the Bonferroni method. Statistical significance was set at* P* < 0.05 and all *P* values were two-sided. All analyses were performed using SPSS Predictive Analytics Software for Windows, version 22.0 (Released 2009; SPSS Inc., Chicago, IL).

## 3. Results

A total of 112 participants were enrolled in this study, including 58 patients in the PN+EN group, 21 patients in the TEN group, and 33 patients in the TPN group. Comparisons of the three groups are shown in Table 1. There were no significant differences in age, sex, smoking history, educational level, or medical history (including hypertension, hyperlipidemia, diabetes, heart disease, cerebrovascular disease, and joint disease) among the three groups (*P* > 0.05). The distribution of alcohol consumption differed significantly among the groups (*P* = 0.042).

The TCM symptoms of spirit disequilibrium were observed 3-4 days after surgery. The comparisons shown in Table 2 revealed that anxiety and dysphoria were more frequently identified in the TEN and PN**+**EN groups than in the TPN group (*P* < 0.05). No significant differences in restlessness, delirious speech, or intolerance were observed in the groups.

Table 3 shows the comparisons of blood indices of the three groups one and seven days after surgery. The levels of LYM, HB, ALB, and PA were significantly higher in participants in the PE+EN group than those in the other groups (all* P* < 0.05), while the WBC and NE counts and HB, PA, and ALB levels were significantly lower in the TPN group than those in the other groups on postoperative day seven (all* P* < 0.05). Comparison of the blood indices on day seven with those on day one after surgery revealed significant changes in all indices in the PE**+**EN group (all* P* < 0.05).

The results of psychological surveys seven days after surgery, including Wong-Banker FPS-R scores, VAS, NRS, SF-36, and HAMA and subscales scores, are shown in Tables 4 and 5. There were significant differences in Wong-Banker FPS-R, VAS, NRS, and HAMA scores among the groups. The scores were significantly lower in the TPN group than those in the other groups (all* P* < 0.05), which indicates a better psychological status. No significant difference was found in the OFBDDSS, self-overall appearance evaluation, and SF-36 scores among the groups.

Table 6 displays the mean hospital length of stay for the participants. The PE+EN group had a significantly shorter length of hospitalization than those of the other groups. The SGA categories indicated that the patients in the PE+EN group had a significantly better nutritional status than those in the other groups (*P* < 0.05). Evaluation of complexion and luster revealed the highest rate of pale complexion in the TPN group on the 13th day after surgery (*P* < 0.05). The levels of K, NA, and CL were significantly lower in Group TEN than those in the other groups (all* P* < 0.05).

## 4. Discussion

The present study assessed the effects of disease and treatment on the quality of life of patients in order to maintain the health of patients after malignant tumor treatment, reduce unnecessary injuries, reduce complications, and ensure their quality of life [2]. However, the traditional methods of feeding are unacceptable for patients with maxillofacial malignant tumors. Because oral and maxillofacial surgery is mostly performed by general anesthesia and tracheal intubation, in order to avoid affecting general anesthesia, tracheal intubation, and field exposure, the gastric tube is not usually placed after the operation, and retention of the gastric tube after operation is a common postoperative nutritional approach for head and neck tumor surgery. Head and neck tumors, especially malignant tumors in the mouth, require the gastric tube to be retained after the operation because of the large surgical area, the wound in the oral cavity, postoperative infection prevention and wound healing, and postoperative dysphagia. After the operation, the intubation of the stomach tube also has a relative risk before the tracheal tube is removed. On one hand, the indwelling of the stomach tube will weaken the pharynx reflex and oral secretions or stomach contents can accumulate in the mouth after anesthesia. However, postoperative swelling of the wound caused the stenosis of the pharynx and the tissue was damaged after the operation, causing hyperemia and edema of the mucous membrane of the nasopharynx and an increased sensitivity to pain. In general, patients with a gastric tube are placed in a semisupine position to reduce diaphragm stimulation by the gastric tube, help the blood flow in the head and neck, reduce wound swelling, and promote wound healing [27]. During gastric tube placement, gastric tube stimulation of the nasopharynx can trigger the stress reaction of the sympathetic nervous and hypothalamic-pituitary systems, causing hemodynamic changes and stimulating the vagus nerve to induce severe nausea, vomiting, tears, and other discomfort. Patients often experience tracheal tube back pressure due to indirect compression of the esophagus wall and tracheal tube on the inner wall of the trachea, causing esophageal cavity stenosis that is detrimental to the passage of the gastric tube [27–29].

In the clinical setting, patients often experience pharyngeal cavity stenosis caused by postoperative swelling, general anesthesia, and pharyngeal edema after tracheal intubation and large defects in the mouth and neck, which can affect swallowing function.

During the process of placing the gastric tube, patients might feel pain and upset, which brings some difficulties in setting up the tube [30]. The antireset tube can cause nasal cavity bleeding in patients, increasing the risk of catheterization. At the same time, oral cancer patients will enter a peak period of wound swelling within 24-72h after skin flap repair, which makes tube placement more difficult, resulting in a sharp increase in blood pressure, muscle strain, and other reactions, which affect the healing of skin flap.

Consideration of the psychological factors is increasing in the field of gastric tube implantation [13]. Incorrect stomach tube insertion causes physical pain as well as great mental tension and psychological fear [31]. Both the physiological and psychological quality of life of the patient should be considered. Unplanned extubation may occur due to slippage of the stomach gastric fixation, patients with irritability, removal of the nasal tube, nausea, frequent vomiting, severe cough causing vomiting, or lack of systematic health education resulting in automatic or passive nasal gastric tube extrusion [32, 33].

In China, clinical nutrition has been established in most hospitals, but the equipment, personnel, and other resources are still not sufficient [34]. Our nutrition support team (NST) are comprised with clinicians, nutritionists, pharmacists, and trained nurses [35]. Upon receiving patient blood reports, these segments work together to adjust the nutritional supporting plans, as long as required by the patient [36]. Clinical nutrition interventions are an indispensable treatment for patients who have undergone oral surgery and an important link in patient prognosis.

The TCM symptoms of spirit disequilibrium observed at 3 or 4 days after surgery manifested as anxiety and upset mental status in the HAMA and subscales scores assessment seven days after surgery revealed that anxiety and dysphoria were most frequently identified in the TEN group and could be earlier identified based on TCM symptoms. The levels of HB, PA, and ALB were the lowest in the TPN group on the seventh day after surgery. In addition, the rate of pale complexion was the highest in the TPN group on the 13th day after surgery. These two observations indicate that lower levels of HB, PA, and ALB may lead to a pale complexion.

Comparison of the psychological status assessment findings between the three patient groups revealed that parenteral nutrition does not affect the external image of the patient, resulted in less suffering, and was more favorable for the patients to accept if it were economically feasible. The cost of parenteral nutrition treatments is high.

Considering the economic feasibility, treatment effects, and follow-up therapies, PN+EN can be an alternative. This combination nutrition can be adjusted for changing situations, resulting in a flexible solution to provide nutritional support to patients and a shorter length of hospitalization. However, this solution requires several days of stomach tube intubation.

## 5. Limitations

First, because of the time required to complete the treatment, most patients were not willing to undergo total enteral nutrition treatments; thus, the sample size of the TEN group was much smaller than those of the other two groups. Second, assessments of psychological status were not conducted before surgery as a baseline due to limited professional resources and poor patient cooperation. Third, additional follow-up studies on the quality of life are necessary to confirm our findings. More studies will be carried on in the future. Finally, our study did not include biomarker data or formal TCM questionnaire (in development); hence, further studies including advanced biomarker tests and formal TCM questionnaires are required.

## 6. Conclusions

As an auxiliary method, TCM symptoms can help identify spirit disequilibrium earlier, which may be associated with blood indices. Despite the high cost and long length of hospitalization, patients who received parenteral nutrition had the best mental status. However, given the considerations of high cost and hospitalization duration, PN+EN may be an alternative, offering a lower cost, shorter length of hospitalization, and satisfactory clinical effects; however, patients require intubation with a stomach tube for several days.

## Figures and Tables

**Table 1:** Baseline characteristics of the three groups before surgery.

**Items**	**PN+EN group (n=58)**	**TPN group (n=33)**	**TEN group (n=21)**	**Test values**	***P *values**
Age (years) (Mean ± SD)	54.50 ± 10.38	57.64 ± 10.33	53.90 ± 9.93	1.220	0.299
Lifestyle					
Smoker					
Ever	15 (13.39%)	9 (8.04%)	3 (2.68%)	6.769	0.343
Occasionally	15 (13.39%)	5 (4.46%)	6 (5.36%)		
Regularly	14 (12.50%)	5 (4.46%)	7 (6.25%)		
Quitted	14 (12.50%)	14 (12.50%)	5 (4.46%)		
Drinking					
Ever	7 (6.25%)	3 (2.68%)	0	11.920	0.042*∗*
Occasionally	35 (31.25%)	20 (17.86%)	17 (15.18%)		
Regularly	4 (3.57%)	0	3 (2.68%)		
Quitted	12 (10.71%)	10 (8.93%)	1 (0.89%)		
Gender, Female	24 (21.38%)	13 (11.61%)	9 (8.04%)	0.114	0.966
Educational level					
≤ 9 years	20 (17.86%)	8 (7.14%)	8 (7.14%)	6.434	0.171
11-12 years	16 (14.29%)	15 (13.39%)	10 (8.93%)		
> 12 years	22 (19.64%)	10 (8.93%)	3 (2.68%)		
Medical History					
Hypertension	10 (8.93%)	4 (3.57%)	3 (2.68%)	0.422	0.937
Hyperlipemia	6 (5.36%)	1 (0.89%)	1 (0.89%)	1.528	0.551
Diabetes	9 (8.04%)	7 (6.25%)	0	5.328	0.076
Heart disease	3 (2.68%)	2 (1.79%)	1 (0.89%)	0.295	0.975
Cerebrovascular Disease	8 (7.14%)	6 (5.36%)	2 (1.79%)	0.763	0.723
Joint disease	4 (3.57%)	3 (2.68%)	0	1.617	0.443
Pathological type					
Malignant ameloblastoma	8 (7.14%)	2 (1.79%)	3 (2.68%)	14.665	0.799
Malignant mixed tumor	4 (3.57%)	5 (4.46%)	1 (0.89%)		
Malignant lymphoma	1 (0.89%)	2 (1.79%)	0		
Ossifying fibroma.	5 (4.46%)	3 (2.68%)	4 (3.57%)		
Basal cell carcinoma	5 (4.46%)	4 (3.57%)	2 (1.79%)		
Oral cancer invade mandible.	10 (8.93%)	3 (2.68%)	6 (5.36%)		
Mucoepidermoid carcinoma	5 (4.46%)	5 (4.46%)	1 (0.89%)		
Adenocarcinoma	2 (1.79%)	1 (0.89%)	1 (0.89%)		
Adenoid cystic carcinoma	3 (2.68%)	2 (1.79%)	1 (0.89%)		
Squamous cell carcinoma	9 (8.04%)	5 (4.46%)	1 (0.89%)		
Odontogenic Keratocyst	6 (5.36%)	1 (0.89%)	1 (0.89%)		

*∗* Significantly different among groups (*P *< 0.05).

Values are demonstrated as n (%) for chi-square or Fisher's exact tests, and as the mean, SD for one-way ANOVA test.

SD: standard deviations.

**Table 2:** TCM symptoms of spirits disequilibrium of the three groups after surgery (3-4 day).

**TCM symptoms**	**PN+EN group (n=58)**	**TPN group (n=33)**	**TEN group (n=21)**	**Test values**	***P *values**
**Anxiety**	6 (5.36%)	2 (1.79%)	6 (5.36%)	5.534	0.044*∗*
**Dysphoria**	2 (1.79%)	2 (1.79%)	5 (4.46%)	7.120	0.018*∗*
**Restlessness**	3 (2.68%)	3 (2.68%)	3 (2.68%)	2.052	0.422
**Delirious speech**	1 (0.89%)	0	1 (0.89%)	1.788	0.426
**Intolerance**	1 (0.89%)	0	1 (0.89%)	1.788	0.426

*∗* Significantly different among groups (*P *< 0.05).

Values are demonstrated as n (%) for chi-square or Fisher's exact tests.

TCM, traditional Chinese medicine.

**Table 3:** Results of the blood indices of the three groups.

**Items**	**Group**	**Postoperative 1 d**	**Postoperative 7d**	**Test values**	***P *values**
WBC	PN+EN	16.13 ±1.81	9.68 ± 1.51	20.358	0.000*∗*
(×10^9^)	TPN	15.51 ± 1.87	7.99 ± 0.94	23.642	0.000*∗*
	TEN	16.73 ± 1.64	10.30 ± 1.37	17.191	0.000*∗*
	**Test values**	2.257	15.661	——	——
	***P *values**	0.113	0.000*∗*	——	——
NE	PN+EN	7.59 ± 1.05	5.81 ± 0.51	10.027	0.000*∗*
(×10^9^)	TPN	7.59 ± 0.94	5.51 ± 0.69	8.168	0.000*∗*
	TEN	7.89 ± 0.75	6.18 ± 0.84	8.312	0.000*∗*
	**Test values**	0.680	4.910	——	——
	***P *values**	0.510	0.010*∗*	——	——
LYM	PN+EN	1.24 ± 0.24	1.82 ± 0.40	7.046	0.000*∗*
(×10^9^)	TPN	1.11 ± 0.32	1.74 ± 0.33	6.394	0.000*∗*
	TEN	1.08 ± 0.31	1.08 ± 0.28	0.013	0.990
	**Test values**	2.277	29.051	——	——
	***P *values**	0.109	0.000*∗*	——	——
HB	PN+EN	116.47 ± 18.00	122.79 ± 12.43	2.385	0.024*∗*
(g/L)	TPN	98.47 ± 5.73	111.17 ± 9.07	4.697	0.000*∗*
	TEN	111.52 ± 12.22	112.35 ± 10.93	0.352	0.728
	**Test values**	9.835	8.181	——	——
	***P *values**	0.000*∗*	0.001*∗*	——	——
PA	PN+EN	252.66 ± 31.68	266.28 ± 23.82	2.777	0.010*∗*
(mg/L)	TPN	230.32 ± 30.59	243.11 ± 38.77	1.370	0.188
	TEN	243.15 ± 33.89	244.70 ± 39.98	0.185	0.855
	**Test values**	2.792	3.695	——	——
	***P *values**	0.069	0.030*∗*	——	——
ALB	PN+EN	38.01 ± 4.00	40.26 ± 2.70	2.716	0.011*∗*
(g/L)	TPN	33.64 ± 3.51	35.12 ± 5.58	1.067	0.300
	TEN	36.02 ± 3.11	37.21 ± 3.57	1.497	0.151
	**Test values**	8.367	10.273	——	——
	***P *values**	0.001*∗*	0.000*∗*	——	——

*∗* Significantly different among groups (*P *< 0.05). Values are demonstrated as the mean ± standard deviations for one-way ANOVA test. The pairwise comparisons among the means were performed using the Bonferroni method.

WBC, white blood cell; NE, neutrophil; LYM, lymphocytes; HB, hemoglobin.

PA, prealbumin; ALB, albumin.

**Table 4:** FPS-R, VAS, NRS, and SF-36 scores of the three groups after surgery (7 day).

**Items**	**PN+EN group (n=58)**	**TPN group (n=33)**	**TEN group (n=21)**	**Test values**	***P *values**
Wong-Banker FPS-R scores	5.0 (4.0, 5.0)	2.0 (2.0, 3.0)	5.0 (4.0, 5.0)	64.205	0.000*∗*
VAS scores	5.0 (5.0, 6.0)	2.0 (2.0, 3.0)	6.0 (6.0, 7.0)	81.016	0.000*∗*
NRS scores	5.0 (5.0, 6.0)	3.0 (2.0, 3.0)	6.0 (5.0, 6.0)	69.809	0.000*∗*
OFBDDSS scores	57.12 ± 14.59	52.58 ± 12.52	53.95 ± 9.52	1.362	0.260
Self - overall appearance evaluation scores	5.50 ± 0.82	5.18 ± 0.77	5.48 ± 0.68	1.858	0.161
SF-36 scores	40.15 ± 3.68	39.00 ± 2.69	40.91 ± 3.36	0.765	0.472

*∗* Significantly different among groups (*P *< 0.05).

Values are demonstrated as n (%) for chi-square or Fisher's exact tests, as the mean ± standard deviations for one-way ANOVA test and as Median, (Quartil _25_, Quartil _75_) for Kruskal-Wails H test.

FPS-R, Faces Pain Scale-Revised; VAS, visual analogue scale; NRS, Numerical rating scale; OFBDDSS, Oral-Facial Body Dysmorphic Disorder Self-Scale; SF-36, short-form 36 health survey questionnaire.

**Table 5:** HAMA and subscales scores of the three groups after surgery (7 day).

**Items**	**PN+EN group (n=58)**	**TPN group** **(n=33)**	**TEN group** **(n=21)**	**Test values**	***P *values**
HAMA scores	14.00 (12.75, 16.00)	10.0 0 (9.00, 12.00)	17.00 (15.00, 17.50)	46.103	0.000*∗*
7-14 scores (patients may have anxiety)	34 (30.36%)	32 (28.57%)	4 (3.57%)	37.854	0.000*∗*
15-21scores (patients definitely have anxiety)	24 (21.43%)	1 (0.89%)	17 (15.18%)		
Anxious state of mind	2.00 (1.00, 2.00)	1.00 (1.00, 1.00)	2.00 (1.00, 2.00)	21.148	0.000*∗*
Tension	2.0 (1.75.0, 2.00)	1.00 (1.00, 1.00)	2.00 (2.00, 2.00)	35.474	0.000*∗*
Fear	2.00 (2.00, 2.00)	1.00 (1.00, 1.00)	2.00 (2.00, 2.00)	45.492	0.000*∗*
Insomnia	1.00 (1.00, 2.00)	1.00 (0.00, 1.00)	1.00 (0.00, 1.00)	22.590	0.000*∗*
Cognitive function	1.00 (0.00, 1.00)	0.00 (0.00, 1.00)	0.00 (0.00, 0.00)	28.728	0.000*∗*
Depressive mood	0.00 (0.00, 0.00)	0.00 (0.00, 1.00)	1.00 (0.00, 1.00)	11.059	0.004*∗*
Muscular system symptoms	1.00 (1.00, 2.00)	1.00 (1.00, 1.00)	1.00 (1.00, 2.00)	15.403	0.000*∗*
Sensation system symptoms	1.00 (1.00, 2.00)	1.00 (1.00, 2.00)	2.00 (1.00, 2.00)	9.124	0.010*∗*
Cardiovascular system symptoms.	0.00 (0.00, 1.00)	0.00 (0.00, 0.00)	1.00 (0.00, 1.00)	14.971	0.001*∗*
Respiratory system symptoms	0.00 (0.00, 1.00)	0.00 (0.00, 1.00)	2.00 (1.50, 2.00)	44.930	0.000*∗*
Gastrointestinal system symptoms	0.00 (0.00, 0.00)	1.00 (1.00, 1.00)	1.00 (0.50, 1.00)	48.342	0.000*∗*
Genitourinary tract symptom	0.00 (0.00, 0.00)	0.00 (0.00, 0.00)	0.00 (0.00, 0.00)	0.377	0.828
Plant nervous system symptoms	1.00 (1.00, 2.00)	1.00 (1.00, 1.00)	1.00 (0.50, 1.00)	17.636	0.000*∗*
Behaviors	2.00 (1.00, 2.00)	1.00 (1.00, 1.00)	2.00 (1.00, 2.00)	12.384	0.002*∗*

*∗* Significantly different among groups (*P *< 0.05). Values are demonstrated as n (%) for chi-square or Fisher's exact tests and as Median (Quartil _25_, Quartil _75_) for Kruskal-Wails H test.

HAMA, Hamilton anxiety rating scale.

**Table 6:** Indexes of the three groups after surgery (13 day).

**Items**	**PN+EN group** ** (n=58)**	**TEN group** **(n=21)**	**TPN group** ** (n=33)**	**Test values**	***P *values**
BMI (kg/m^2^)	22.13 ± 1.92	21.67 ± 2.65	21.64 ± 2.06	0.711	0.493
Length of hospitalization (days)	16.90 ± 3.81	21.15 ± 3.12	21.95 ± 3.17	15.344	0.000*∗*
K (mmol/L)	4.10 ± 0.33	3.25 ± 0.56	4.29 ± 0.29	46.912	0.000*∗*
Na (mmol/L)	133.84 ± 5.40	128.94 ± 9.73	135.26 ± 7.27	4.053	0.022*∗*
CL (mmol/L)	107.85 ± 4.23	103.34 ± 3.56	107.33 ± 5.15	7.077	0.002*∗*
SGA categories					
A grade	9 (8.04%)	0	0	28.139	0.000*∗*
B grade	46 (41.07%)	20 (17.86%)	11 (9.82%)		
C grade	3 (2.68%)	13 (11.61%)	10 (8.93%)		
TCM symptoms					
Pale complexion	1 (0.89%)	2 (1.79%)	6 (5.36%)	11.859	0.001*∗*
Skin without luster	7 (6.25%)	3 (2.68%)	5 (4.46%)	2.451	0.280

*∗* Significantly different among groups (*P *< 0.05). Values are demonstrated as n (%) for chi-square or Fisher's exact tests, as the mean ± standard deviations for one-way ANOVA test.

Abbreviations: BMI, body mass index; K, potassium; NA, sodium; CL, chlorine; SGA, subjective global assessment; TCM, traditional Chinese medicine.

## Data Availability

This study was registered at the Chinese Clinical Trial Registry (http://www.chictr.org.cn) as ChiCTR-OPC-17013869. All the results will be uploaded to the net when the study subject is finished.

## References

[1] Chen W. L., Li J. S., Yang Z. H., Huang Z. Q., Wang J. Q. (2009). Extended vertical lower trapezius island myocutaneous flap for repairing extensive oropharyngeal defects. *Journal of Oral and Maxillofacial Surgery*.

[2] Gondivkar S. M., Gadbail A. R., Sarode S. C., Patil S. (2017). Quality of life assessment should be part of oral health evaluations in day-to-day practice. *Journal of Contemporary Dental Practice*.

[3] Chen H. Y., Wu D. B., Peng Y. L., Meng Y. S., Yang H. Y. (2013). Psychological analysis and nursing intervention for perioperative patients with mouth cancer. *Chinese Journal of Modern Nursing*.

[4] Brüggemann P., Szczepek A. J., Klee K., Gräbel S., Mazurek B., Olze H. (2017). In patients undergoing cochlear implantation, psychological burden affects tinnitus and the overall outcome of auditory rehabilitation. *Frontiers in Human Neuroscience*.

[5] Bissinger O., Rau A., Koerdt S., Wolff K. D., Kesting M. R., Götz C. (2017). Evaluating tumour after care in oral squamous cell carcinoma: Insights into patients' health related quality of life. *Journal of Cranio-Maxillo-Facial Surgery*.

[6] Sroussi H. Y., Epstein J. B., Bensadoun R. J. (2017). Common oral complications of head and neck cancer radiation therapy: mucositis, infections, saliva change, fibrosis, sensory dysfunctions, dental caries, periodontal disease, and osteoradionecrosis. *Cancer Medicine*.

[7] Sahni V. (2018). Psychological impact of facial trauma. *Craniomaxillofacial Trauma & Reconstruction*.

[8] Baumeister H., Härter M. (2011). Psychological comorbidity in patients with musculoskeletal diseases. *Bundesgesundheitsblatt - Gesundheitsforschung - Gesundheitsschutz*.

[9] Cui Y., Liao S., Wang H. W. (2015). ROC-boosting: a feature selection method for health identification using tongue image. *Computational and Mathematical Methods in Medicine*.

[10] Cui Y., Liao S., Wang H., Liu H., Wang H. W., Yin L. Q. (2015). Relationship between hyperuricemia and haar-like features on tongue images. *BioMed Research International*.

[11] Zhou X., Xu F., Gao J. (2015). Development and preliminary validation of the questionnaire (the first edition) based on tcm for detecting health status in China. *Evidence-Based Complementary and Alternative Medicine*.

[12] Zhou X., Cao S., Feng X. Y., Zhao Z. W., Wang H. W. (2016). Theory of essence, qi, and spirit combined with pyramid-like solid structure to explore the health status of human body. *Journal of Tianjin University of Traditional Chinese Medicine*.

[13] Bishop S., Reed W. M. (2015). The provision of enteral nutritional support during definitive chemoradiotherapy in head and neck cancer patients. *Journal of Medical Radiation Sciences*.

[14] Zou C., Zhou X., Liu M., Huang G. W. (2018). The Influence of different nutrition treatment approaches in the fibula muscle flap reconstruction of the mandibular defect postoperative patients. *Parenteral & Enteral Nutrition*.

[15] Ware L. J., Epps C. D., Herr K., Packard A. (2006). Evaluation of the revised faces pain scale, verbal descriptor scale, numeric rating scale, and iowa pain thermometer in older minority adults. *Pain Management Nursing*.

[16] Vittayakittipong P. (2013). Donor-site morbidity after fibula free flap transfer: A comparison of subjective evaluation using a visual analogue scale and point evaluation system. *International Journal of Oral and Maxillofacial Surgery*.

[17] Oxberry S., Middleton V., Bennett M. (2005). What does a good quality of life for cancer patients mean on a numerical rating scale?. *Clinical Oncology*.

[18] Bijur P. E., Latimer C. T., Gallagher E. J. (2003). Validation of a verbally administered numerical rating scale of acute pain for use in the emergency department. *Academic Emergency Medicine*.

[19] Thompson E. (2015). Hamilton rating scale for anxiety (HAM-A). *Occupational Medicine*.

[20] Cai Z., Dai F. (2004). The design of self-assessment scale and clinical application of orthodontics in the oral and maxillofacial features. *Chinese Journal of Orthodontics*.

[21] Lu L. G., Chen T. N., Chen J. G., Lin W. G. (2000). A self rating scale of body image. *Chinese Mental Health Journal*.

[22] Ma Y., Yin Z. M., Lu Y., Hao S. W., Xu Z. L. (2015). The difference between self evaluation and other evaluation of facial beauty and impact of self-esteem on it. *Chinese Journal of Health Psychology*.

[23] Deng N., He W., Li R., Li W. L., Gao N., Zhang W. (2015). Assessment of the quality of life of oral cancer patients after reconstruction with free anterolateral thigh perforator flaps. *West China journal of stomatology*.

[24] Al-Ahmad H. T., Al-Bitar Z. B. (2014). The effect of temporomandibular disorders on condition-specific quality of life in patients with dentofacial deformities. *Oral Surgery, Oral Medicine, Oral Pathology, Oral Radiology, and Endodontology*.

[25] Kuzu M. A., Terzioğlu H., Genç V. (2006). Preoperative nutritional risk assessment in predicting postoperative outcome in patients undergoing major surgery. *World Journal of Surgery*.

[26] Anthony P. S. (2008). Nutrition screening tools for hospitalized patients. *Nutrition in Clinical Practice*.

[27] Motsch C., Kahl S., Nebelung K. (2001). Fundamentals of enteral feeding, tube techniques, PEG. *Laryngo-Rhino-Otologie*.

[28] Geng S. X. (2009). Clinical study of difficulties in stomach intubation method. *China Practical Medicine*.

[29] DeLegge M. H. (2018). Enteral access and associated complications. *Gastroenterology Clinics of North America*.

[30] Merrick S., Farrell D. (2012). Head and neck cancer patients' experiences of percutaneous endoscopic gastrostomy feeding: A Q-methodology study. *European Journal of Cancer Care*.

[31] Penrod J., Morse J. M., Wilson S. (1999). Comforting strategies used during nasogastric tube insertion. *Journal of Clinical Nursing*.

[32] Chen A. P., Cai M. (2007). Unplanned extraction and related research progress of ICU patients. *Chinese Journal of Nursing*.

[33] McCall M. E., Adamo A., Latko K., Rieder A. K., Durand N., Nathanson T. (2018). Maximizing nutrition support practice and measuring adherence to nutrition support guidelines in a canadian tertiary care ICU. *Journal of Intensive Care Medicine*.

[34] Wang J., Zuo X. X., Zhang Y., Shen T. T. (2015). The present situation and improvement strategy of clinical nutrition department in hospital in China. *People's Military Surgeon*.

[35] Wang J., Zhao Y. R. (2017). The role of clinical nutrition department in clinical treatment. *Parenteral & Enteral Nutrition*.

[36] Wong A., Goh G., Banks M. D., Bauer J. D. (2018). A systematic review of the cost and economic outcomes of home enteral nutrition. *Clinical Nutrition*.

